# Redescription of *Dracovermis occidentalis* (Digenea: Liolopidae) infecting American alligator, *Alligator mississippiensis* from the Bon-Secour River (Mobile–Tensaw River Delta, Alabama, USA) and a revised phylogeny for Liolopidae

**DOI:** 10.1007/s00436-024-08339-2

**Published:** 2024-09-19

**Authors:** Haley R. Dutton, Stephen A. Bullard, John H. Brule, Anita M. Kelly

**Affiliations:** 1 Southeastern Cooperative Fish Parasite and Disease Laboratory, School of Fisheries, Aquaculture, and Aquatic Sciences, College of Agriculture, Auburn University, 559 Devall Drive, Auburn, Al 36849 USA; 2 Unit for Environmental Sciences and Development, North-West University, Private Bag X6001, Potchefstroom, 2520 South Africa; 3 Alabama Fish Farming Center, School of Fisheries, Aquaculture, and Aquatic Sciences, College of Agriculture, Auburn University, Greensboro, AL 36744 USA

**Keywords:** Parasite, Digenean, Taxonomy, Systematics, Reptile

## Abstract

We examined several American alligators, *Alligator mississippiensis* (Daudin, 1802) (Crocodilia: Alligatoridae) from Louisiana, Alabama, and South Carolina in August 2022. The intestine of one alligator from Alabama was infected by *Dracovermis occidentalis* Brooks and Overstreet, 1978 (Platyhelminthes: Digenea: Liolopidae Odhner, 1912), a seldom collected and incompletely described trematode that lacks a representative nucleotide sequence. Liolopidae comprises 5 genera and 15 species: *Liolope* spp. infect giant salamanders; *Helicotrema* spp. infect turtles and lizards; *Harmotrema* spp. infect snakes; *Paraharmotrema* spp. infect turtles; and *Dracovermis* spp. infect crocodilians. Based on our study of the newly collected specimens and the holotype of *D. occidentalis*, we redescribe *D. occidentalis*, correct errors in its original description, and provide an updated phylogeny for Liolopidae that, for the first time, includes *Dracovermis* Brooks and Overstreet, 1978. Our specimens were identified as *D. occidentalis* by having testes in the posterior 1/3 of the body, a pretesticular cirrus sac, a spined and eversible cirrus, a bipartite seminal vesicle, and a post-acetabular vitellarium. A phylogenetic analysis of the D1–D3 domains of the nuclear large subunit ribosomal DNA (*28S*) recovered Liolopidae as monophyletic; however, low taxon sampling in the group precludes hypothesis-testing about liolopid-vertebrate cophyly. This is the first collection for morphological study of the type species for *Dracovermis* since the genus was proposed 46 years ago, the first record of a liolopid from Alabama, and the first phylogenetic analysis that includes *Dracovermis*.

## Introduction

The American alligator, *Alligator mississippiensis* (Daudin, 1802) (Crocodilia: Alligatoridae), hereafter "alligator", is the largest predatory reptile in North America. Historically abundant and ranging across coastal/estuarine waters and semi-inland riverine systems throughout the southeastern USA, alligators were hunted/poached for their skin and nearly driven to extinction in the early 1900s. They remained considered threatened as late as the 1960s but proper management has allowed alligator populations to recover relatively rapidly, with the US Fish and Wildlife Service in 1979 downlisting the alligator from CITES Appendix I to Appendix II; allowing for some legal international trade of alligator skins (Ashley and Caldwell 2013; Elsey and Woodward 2010; Jelden et al. 2014; PIJAC 2013; Thorbjarnarson et al. 1992). Regarding captive stocks of alligators, a survey conducted in 2013 revealed that > 100 commercial farms in the US maintained ~ 790,000 alligators, with 20,000 hatchlings being produced from these facilities annually. Based on these facts, along with the cultural and conservation value of alligators in the wild, as well as the commercial value of the large standing stock of alligators in aquaculture ponds, we assert that infectious diseases and parasites are relevant to alligator population health and captive husbandry/maintenance. This is in addition to the general interest in parasites of crocodilians regarding parasite-host cophyly and crocodilian natural history.

Although alligators are among the most iconic and well-studied reptiles in North America regarding their life history and general biology, studies on the taxonomy and life cycles of their parasites have been relatively limited. This could be in part due to the difficulty in obtaining permission to sample alligators legally, logistical challenges associated with dissecting and processing large alligators in the field, as well as the technical challenges of extracting, handling, and fixing live, minute trematodes in the field. During a state-sponsored alligator hunt in coastal Alabama, we necropsied fresh-killed alligators during the hunter registration of alligator carcasses with natural resource agency personnel. From those collections, we herein provide a redescription for *Dracovermis occidentalis* Brooks and Overstreet 1978 and place it in a phylogenetic analysis for the first time using the large subunit ribosomal DNA (*28S*) to test relationships and monophyly of the Liolopidae Odhner, 1912.

## Materials and methods

Alligators were sampled at a coastal registration station (30°40′19.3"N 87°56′06.6"W) operated by the Alabama Division of Wildlife & Freshwater Fisheries for the annual Alabama alligator season hunt during August 2022. We encountered *D. occidentalis* infecting the intestine of 1 alligator. The infected male alligator (Tag 1329) measured 2.52 m long and was caught by a baited line in the Bon Secour River, Alabama, by Jarrod Pettie. The digestive tract was excised intact, sliced longitudinally to expose the lumen, rinsed in saline, and decanted and placed into acrylic settling columns before the sediment was observed in a petri dish under the dissecting scope and examined for live flukes.

Trematodes intended for morphology as whole-mounts were observed microscopically and fixed following Dutton et al. (2022). Whole mounts were examined and illustrated using an Olympus BX53 microscope (Olympus Corporation, Shinjuku City, Tokyo, Japan) equipped with differential interference contrast, measured using an ocular micrometer, and illustrated using a drawing tube. Measurements are reported in micrometers (μm) as the range followed by the mean, + / − standard deviation, and sample size in parentheses. Measurements of the holotype (USNM 1370158) are provided in brackets. Vouchers were deposited in the National Museum of Natural History’s Invertebrate Zoology Collection (Smithsonian Institution, USNM Collection Nos. 1718009, 1718010, 1718012–1718015). Classification and anatomical terms for liolopids follow Brooks and Overstreet (1978) and Dutton et al (2022).

Total genomic DNA was extracted from 1 EtOH-preserved specimen. The specimen was microscopically identified by the presence of the cirrus spines and genitalia in posterior third of the body as *D. occidentalis*. The specimen was extracted using DNeasy™ Blood and Tissue Kit (Qiagen, Hilden, Germany) as per the manufacturer’s protocol, except that the proteinase-K incubation period was extended overnight, and 50 μL of elution buffer was used to increase the final DNA concentration. Amplification and sequencing of the *28S*, *18S*, *ITS2* and the *COX1* used the following primer sets: *28S* (LSU-5 [5′-TAGGTCGACCCGCTGAAYTTAAGCA-3′] and 1500R [5′-GCTATCCTGAGGGAAACTTCG-3′]), *18S* (WormA [5′-GCGAATGGCTCATTAAATCAG-3′] and Worm B [5′-CTTGTTACGACTTTTACTTCC-3′] [Littlewood and Olson 2014]), *ITS2* (GA1 [5′-AGAACATCGACATCTTGAAC-3′] [Anderson and Barker 1998] and ITS2.2 [5′-CCTGGTTAGTTTCTTTTCCTCCGC-3′] [Cribb et al. 1998]), *COX1* (JB3 [5′-TTTTTTGGGCATCCTGAGGTTTAT-3′] [Bowles and McManus, 1993] and CO1-R trema [5′-CAACAAATCATGATGCAAAAGG-3′] [Miura et al., 2005]). PCR reactions were performed with a total volume of 50 μL (34 μL purified water, 10 μL 5X Taq buffer [Promega Corporation, Madison, Wisconsin], 1 μL 10 mM dNTP [Promega Corporation], 1 μL 10 mM forward primer, 1 μL 10 mM reverse primer, 0.3 μL GoTaq DNA polymerase [Promega Corporation], and 2 μL DNA extract). The thermocycling profile for the *28S* comprised 4 min at 94 ºC for denaturation, 40 repeating cycles at 94 ºC for 40 s for denaturation, 56 ºC for 30 s for annealing and 72 ºC for 2 min for extension followed by a final 5 min at 72 ºC for extension. The *18S* and *ITS2* follow the same profile except 50 ºC for 30 s for annealing, and 72 ºC for 1 min for extension. The *COX1* thermocycling profile comprised 2 min at 94 ºC for denaturation, 40 repeating cycles at 94 ºC for 30 s for denaturation, 52 ºC for 30 s for annealing, and 72 ºC for 2 min for extension followed by a final 7 min at 72 ºC for extension. All PCR reactions were carried out in a ProFlex PCR System (Applied Biosystems, Waltham, MA). PCR products (10 μL) were verified on a 1% agarose gel and stained with ethidium bromide. PCR products were purified with the QIAquick PCR Purification Kit (Qiagen) according to the manufacturer’s protocols except that the last elution step was performed with autoclaved nanopure H2O rather than with the provided buffer. Purified DNA concentration was estimated using a NanoDrop™ One Microvolume UV–Vis Spectrophotometer (Thermo Fisher Scientific Inc., Waltham, Massachusetts). Purified DNA samples were prepared for sequencing with a total volume of 15 ml (2 μL 10 mM primer + purified DNA + purified water) with volumes of purified DNA and water depending on DNA sample concentration. DNA sequencing was conducted by GENEWIZ (Azenta Life Sciences, South Plainfield, New Jersey). All assembled contiguous nucleotide sequences were deposited in GenBank (PQ186364, PQ186366, PQ186369, PQ187446).

The *28S* phylogenetic analysis included the single sequence from the current study and the other taxa used in Dutton et al. (2022). Sequences were aligned with the multiple alignment tool using fast Fourier transform (MAFFT) (Katoh and Standley 2013) and trimmed to the length of the shortest sequence presented herein (1,237 base pairs [bp]) in Geneious Prime Software version 2023.0.4 (Geneious Corp., Auckland, New Zealand). Aligned sequences were reformatted and exported from.fasta to.phy to run a maximum likelihood tree (ML). The ML was inferred with IQTREE v.1.16.12 (Nguyen et al. 2015). Substitution model testing was done with ModelFinder (Kalyaanamoorthy et al. 2017) as implemented in IQTREE. After model testing, tree inference was done using best-fitting substitution models (Chernomor et al. 2016). Default tree search parameters were used, except perturbation strength was set to 0.2, and 500 iterations had to be unsuccessful to stop the tree search. Tree inference was performed 20 times with only the tree with the best log-likelihood score reported. Support for relationships was measured with 1000 ultrafast bootstrap replicates (UFBoot) (Hoang et al. 2018). The inferred phylogenetic tree was visualized using FigTree v1.4.4 (Rambaut et al. 2018) and further edited with Adobe Illustrator (Adobe Systems) for visualization purposes. With only 4 sequences available for the *28S*, 2 sequences available for the *18S* and *ITS2*, and 1 sequence for the *CO1* we have chosen not to run the individual trees for each region but continue to create a genetic library for future use.

## Results

### Dracovermis occidentalisBrooks and Overstreet 1978 emend. Dutton and Bullard,2024 (Figs. 1 and 2)

**Fig. 1:**
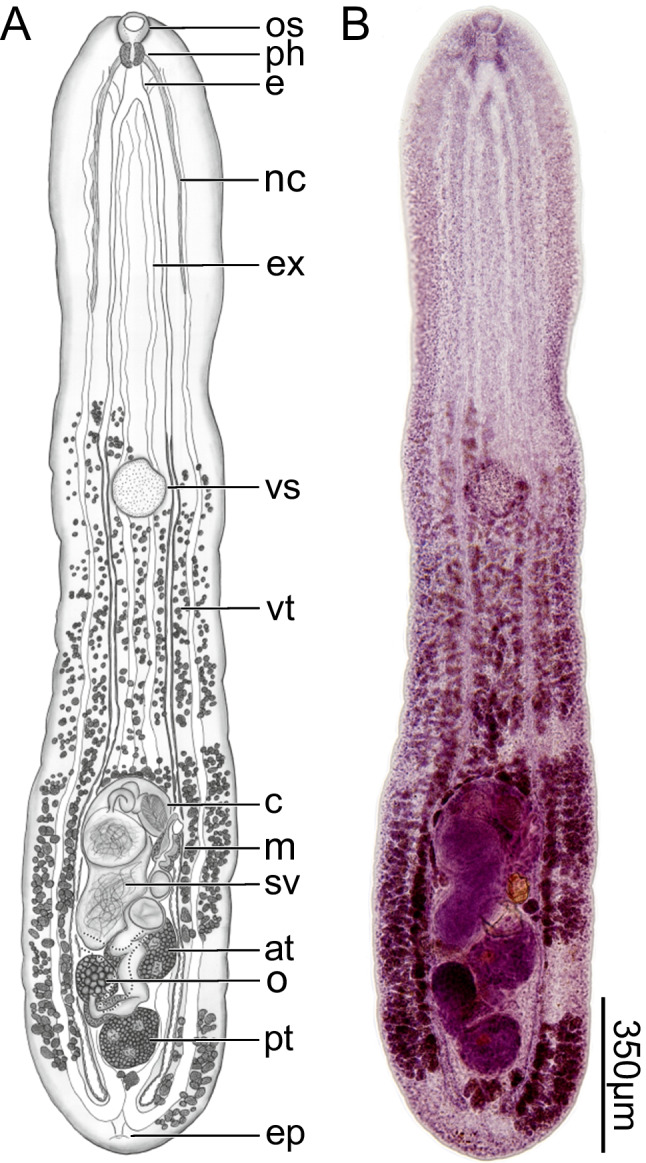
*Dracovermis occidentalis* Brooks and Overstreet 1978 emend. Dutton and Bullard, 2024 (Digenea: Liolopidae Odhner, 1912) infecting intestine of American alligator, *Alligator mississippiensis* Daudin, 1802 (Crocodilia: Alligatoridae) from Mobile Bay, Alabama. Scale values aside bars. (A) Body of voucher (USNM No. 1718009) showing oral sucker (os), pharynx (ph), esophagus (e), nerve cord (nc), excretory system (ex), ventral sucker (vs), vitellarium (vt), cirrus (c), metraterm (m), seminal vesicle (sv), anterior testis (at), ovary (o), posterior testis (pt), and excretory pore (ep). Ventral View. (B) Light micrograph body of voucher (USNM No. 1718009). Ventral View

**Fig. 2:**
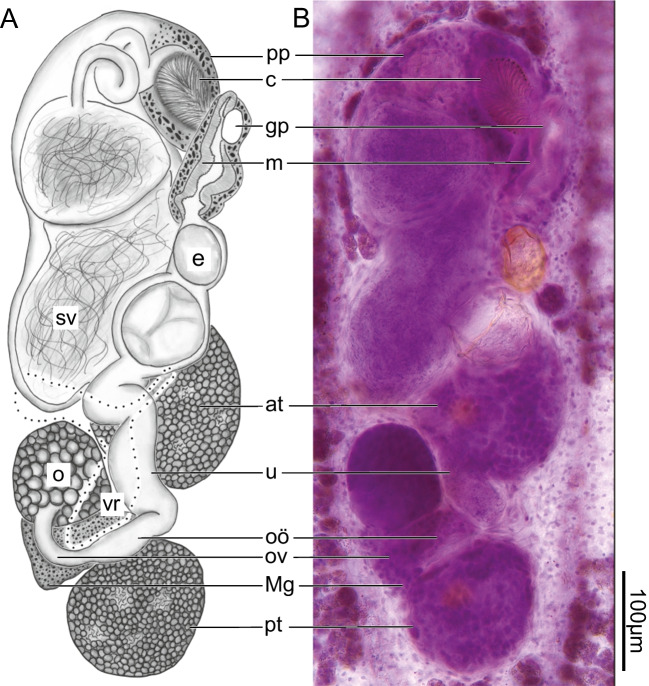
*Dracovermis occidentalis* Brooks and Overstreet 1978 emend. Dutton and Bullard, 2024 (Digenea: Liolopidae Odhner, 1912) infecting intestine of American alligator, *Alligator mississippiensis* Daudin, 1802 (Crocodilia: Alligatoridae) from Mobile Bay, Alabama. Scale values aside bars. (A) Genitalia of voucher (USNM No. 1718009) showing pars prostatica (pp), cirrus (c), genital pore (gp), metraterm (m), egg (e), seminal vesicle (sv), anterior testis (at), ovary (o), uterus (u), vitelline reservoir (vr), oötype (oö), oviduct (ov), Mehlis’ gland (Mg), and posterior testis (pt). Ventral View. (B) Light micrograph genitalia of voucher (USNM No. 1718009) Ventral View

Based on light microscopy of 6 heat-killed, stained whole-mounted adult specimens (USNM 1718009, 1718010, 1718012–1718015) and the holotype (USNM 1370158)

Body linguiform in heat-killed specimens (Fig. 1), pyriform or slightly ovoid in outline in holotype, 2,125 − 2,965 (2,659 ± 327; 6) [1,150] long, 370 − 650 (536 ± 98; 6) [480] in maximum width at the level of genital pore, 4 − 6 × (5 ± 1; 6) [2.4 ×] longer than wide, with clear ventral concavity only observed in specimens having ventrally curved edges; forebody (anterior to ventral sucker) 35 − 47% (40% ± 5%; 6) [30%] of total body length, spinose; forebody spines scattered about ventral surface of forebody [absent or indistinct in holotype, perhaps related to poor condition of specimen]; body surfaces lacking papillae. Oral sucker bowl shaped, 43 − 69 (57 ± 12; 5) [45] long or 2 − 3% (2% ± 0%; 5) [4%] of body length, 57 − 80 (72 ± 10; 5) [50] wide or 10 − 15% (13% ± 2%; 5) [10%] of maximum body width, 1: 1.1 − 1.5 × (1.3 ± 0.2; 5) [1.8 ×] pharynx width, aspinose. Ventral sucker thin and delicate (Fig. 1), 135 − 160 (143 ± 9; 6) [125] long or 5 − 6% (5% ± 1%; 6) [11%] of body length, 120 − 141 (132 ± 8; 6) [115] wide or 36 − 48% (43% ± 6%; 4) [38%] of maximum body width; 1.8 − 2.2 × (1.9 ± 0.2; 5) [2.3 ×] oral sucker width. Nerve commissure 87 − 120 (103 ± 13; 5) [70] or 3 − 4% (4% ± 0%; 5) [6%] of body length from anterior body end. Pharynx 55 − 84 (68 ± 11; 5) [43] long, 49 − 70 (56 ± 8; 5) [28] wide. Esophagus short 45 − 150 (86 ± 45; 4) long, 13 − 20 (16 ± 4; 4) wide; esophageal gland absent. Intestine comprising a cecal bifurcation and paired ceca, each extending posteriad approximately in parallel with respective lateral body margin, blind ending in posterior body end, symmetrical; cecal bifurcation 120 − 231 (172 ± 51; 4) [70] or 5 − 8% (6% ± 1%; 4) [6%] of body length from anterior body end; wall of ceca thickening markedly at level immediately anterior to ventral sucker (Fig. 1).

Anterior testis (Figs. 1, 2) 110 − 175 (148 ± 26; 6) [118] long or 4 − 6% (6% ± 1%; 6) [10%] of body length, 125 − 217 (179 ± 33; 6) [188] wide or 24 − 51% (34% ± 9%; 6) [39%] of body width at level of genital pore or 60 − 146% (110% ± 30%; 6) [130%] of posterior testis width; inter-testicular space 15 − 113 (71 ± 34; 6) [33] long or 1 − 4% (3% ± 1%; 6) [3%] of body length; posterior testis 115 − 225 (167 ± 39; 6) [133] long or 5 − 8% (6% ± 1%; 6) [12%] of body length, 130 − 210 (168 ± 30; 6) [145] wide or 27 − 40% (32% ± 5%; 6) [30%] of body width at level of genital pore, 125 − 197 (163 ± 29; 6) [75] or 5 − 7% (6% ± 1%; 6) [7%] of body length from posterior body end; anterior trunk of vasa efferentia emanating from ventral surface of anterior testis, extending directly into posterior end of seminal vesicle; posterior trunk of vasa efferentia emanating from ventral surface of posterior testis, extending anteriad 125 − 275 (203 ± 75; 3) [155], seemingly connecting to seminal vesicle directly (vas deferens indistinct). Cirrus sac elongate, slightly curved (“semilunar”) (Figs. 1, 2), 305 − 610 (496 ± 106; 6) [240] long or 14 − 21% (18% ± 2%; 6) [21%] of body length, 295 − 561 (443 ± 114; 6) [188] wide or 10 − 23% (17% ± 5%; 6) [39%] body width at level of genital pore; internal seminal vesicle bipartite, comprising proximal and distal portions; proximal portion of seminal vesicle 105 − 270 (186 ± 81; 6) [125] long or 4 − 11% (7% ± 3%; 6) [11%] of body length, 46 − 176 (125 ± 45; 6) [65] wide, 1 × (1 ± 0; 6) [0.52 ×] longer than wide; distal portion of seminal vesicle ovoid in outline, 100 − 161 (133 ± 22; 6) [100] long or 4 − 7% (5% ± 1%; 6) [9%] of body length, 45 − 145 (112 ± 36; 6) [100] wide, 1 × (1 ± 0; 6) [1 ×] longer than wide; cirrus comprises a coiled duct (“ejaculatory duct” of Brooks and Overstreet [1978]) that leads to spined cirrus (Figs. 1,2), 75 − 132 (105 ± 20; 6) [75] long or 14 − 37% (25% ± 8%; 6) [31%] of cirrus sac length, 53 − 130 (75 ± 28; 6) [73] wide; cirrus spines curved, tapering distally, 28 − 37 (31 ± 4; 7) [25] long; pars prostatica surrounding cirrus; common genital atrium present.

Ovary entire (Figs. 1, 2), 84 − 140 (111 ± 18; 6) [95] long or 3 − 5% (4% ± 1%; 6) [8%] of body length, 93 − 130 (111 ± 15; 6) [95] wide or 14 − 34% (22% ± 7%; 6) [20%] of body width; post-ovarian space 225 − 405 (357 ± 68; 6) [200] or 11 − 15% (13% ± 1%; 6) [17%] of body length. Oviduct 43 − 123 (98 ± 30; 6) [88] long or 2 − 5% (4% ± 1%; 6) [8%] of body length, 38 − 66 (47 ± 11; 6) [50] wide, posterior to transverse vitelline duct, laterally expanding before becoming confluent with oötype. Laurer's canal not observed. Oötype 50 − 107 (70 ± 22; 6) long, 20 − 63 (39 ± 18; 6) wide, between ovary and posterior testis. Uterus convoluted, 255 − 684 (415 ± 154; 6) [413] long, 33 − 115 (75 ± 31; 6) [75] wide; uterine seminal receptacle present; uterine eggs elongate, 70 − 122 (99 ± 23; 6) [113] long, 28 − 80 (61 ± 19; 6) [75] wide, numbering 1 − 7 (4 ± 3; 6) [1] per specimen. Metraterm 158 − 230 (178 ± 27; 6) [100] long, 31 − 117 (67 ± 31; 6) [63] wide, thick walled, surrounded by small glandular cells; total uterus + metraterm length 413 − 914 (586 ± 179; 6) [350] or 18 − 31% (22% ± 5%; 6) [30%] of body length. Common genital pore 485 − 872 (728 ± 131; 6) [450] or 23 − 36% (27% ± 5%; 6) [39%] of body length from posterior body end, 38 − 70 (54 ± 13; 6) [20] in diameter. Vitellarium comprising a series of small, irregular shaped follicles, distributing along ceca and excretory branches from level of ventral sucker or slightly anterior to end of body (Fig. 1); confluence of vitellarium 7 − 70 (28 ± 29; 6) [58] or 0 − 3% (1% ± 1%; 6) [5%] from posterior body end; transverse vitelline duct dorsal, 245 − 426 (307 ± 67; 6) [225] in breadth, 15 − 41 (25 ± 11; 6) [38] wide; primary vitelline collecting duct 40 − 147 (100 ± 45; 6) [73] long, 45 − 75 (56 ± 10; 6) [38] wide, anterior to oötype. Excretory system having extracecal and intercaecal tubules (Fig. 1), 1,970 − 2,744 (2,481 ± 312; 6) [1050] long; intracecal branches joining at level of cecal tips; excretory pore subterminal.

### Taxonomic summary

*Type and only known host:* American alligator, *Alligator mississippiensis* (Daudin, 1802) (Crocodilia: Alligatoridae).

*Type locality:* Cameron Parish, Louisiana, USA.

*Other localities:* Horn Island, Jackson County, Mississippi, USA; Chambers, Jefferson, and Liberty counties in southeast Texas, USA; Bon Secour River (present study), Alabama, USA.

*Prevalence and intensity of infection:* 1 of 8 (20%) alligators from Alabama were infected by 7 specimens of *D. occidentalis*, 0 of 15 (0%) alligators from Rockefeller Wildlife Refuge, Cameron Parish, Louisiana, and 0 of 20 (0%) alligators from South Carolina were infected with *D. occidentalis*.

*Specimens deposited*: Vouchers (USNM 1718009, 1718010, 1718012–1718015).

*Other specimens examined:* Holotype USNM 1370158.

*Site of infection:* Intestine.

## Taxonomic remarks

Our specimens were identified as *D. occidentalis* by having testes in the posterior 1/3 of the body, a pretesticular cirrus sac, a spined and eversible cirrus, a bipartite seminal vesicle, and a post-acetabular vitellarium. While not all of these features were clearly described in its original description, we confirmed the presence of these features in our newly collected specimens and the holotype. Noteworthy also is that our specimens of *D. occidentalis* were collected adjacent to the type locality for this species in the northern Gulf of Mexico.

Brooks and Overstreet’s (1978) specimens were contracted upon fixation, whereas ours were heat-killed and formalin-fixed, and we herein correct several errors related to *D. occidentalis* in Brooks and Overstreet (1978) that were due to the poor quality of the types. First, Brooks and Overstreet (1978) stated that the body surface of *Dracovermis* spp. lacks spines; however, in our specimens of *D. occidentalis* we observed spines scattered about the ventral surface of the forebody. These spines were absent or indistinct in the holotype of *D. occidentalis*, perhaps related to poor specimen condition. Second, the body shape of *Dracovermis* spp. is elongate, having equally broadly rounded anterior and posterior ends. This pattern is obscured in the original published description of *D. occidentalis* because of the poor quality of the types. The body shape of *D. occidentalis* depicted in Brooks and Overstreet (1978) is misleading in that the body appears compact and pyriform (similar to many other trematodes), not elongate and linguiform like in other heat-killed specimens of Liolopidae. Our heat-killed and formalin fixed specimens of *D. occidentalis* demonstrate the natural habitus of the species. Third, the ventral sucker is relatively thin and delicate with muscle fibers visible along the periphery of the sucker. Brooks and Overstreet (1978) erroneously stylized the ventral sucker as being thick-walled and extensively muscular, when in fact, the sucker appears similar to that of other liolopids. Fourth, the metraterm in heat-killed and formalin-fixed specimens is straight and strongly muscular. The holotype of *D. occidentalis* is strongly contracted such that the metraterm presents as a compressed coil, whereas in heat-killed specimens the metraterm is straight because the specimen is not contracted. Brooks and Overstreet (1978) diagnosed *D. occidentalis* as having a "folded metraterm." Fifth, the vitellarium in our specimens of *D. occidentalis* is patchily distributed, having gaps among clusters of vitelline follicles. Brooks and Overstreet (1978) highly stylized the vitellarium as a contiguous and evenly distributed field. Although typical for some trematode groups, it is taxonomically significant that the vitellarium of *D. occidentalis* is patchy (not evenly distributed) and can be confluent posteriorly or not confluent posteriorly. Lastly, we were unable to measure the esophagus and oötype in the type specimen due to the contracted nature of the holotype specimen.

Dutton et al. (2022) omitted the following species from their table listing records of liolopids: *Harmotrema indica* Chattopadhyaya, 1970 from “*Enhydrina schistosa*” (= *Hydrophis schistosus* Daudin, 1803) (Squamata: Elapidae) from off of the coast of Bombay, Mumbai, India (Chattopadhyaya, 1970); *Harmotrema microrchis* Bhutta and Khan 1975 from *Gavialis gangeticus* (Gmelin, 1789) (Crocodilia: Gavialidae) from the Sutlej River, Pakistan (Bhutta and Khan 1975); and *Harmotrema linguiforme* Wang 1987 from *Hydrophis cyanocinctus* Daudin, 1803 (Squamata: Elapidae) from Fujian, China (Wang 1987).

## Phylogenetic analysis

**Fig. 3:**
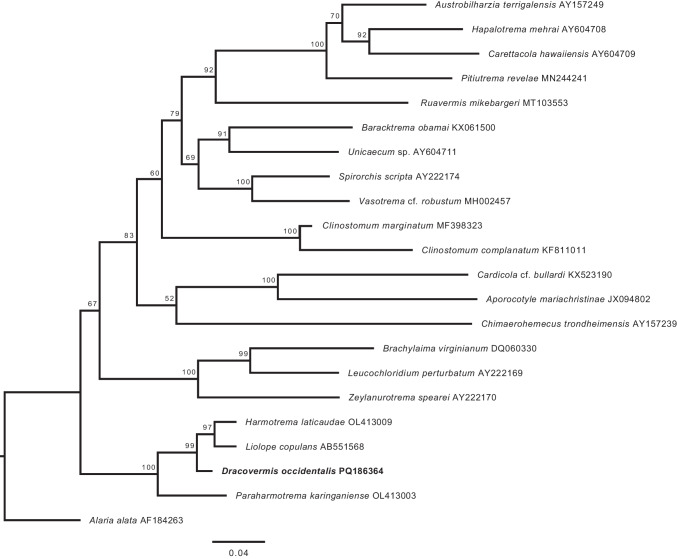
Large subunit ribosomal (*28S*) DNA phylogeny (Maximum Likelihood). Values aside nodes are ultrafast bootstrap replicates (UFBoot). Scalebar indicates the number of substitutions per site. *Dracovermis occidentalis* Brooks and Overstreet 1978 emend. Dutton and Bullard, 2024 is shown in bold and GenBank accession numbers follow each taxon

Our *28S* sequence of *D*. *occidentalis* comprised 1,270 nucleotides (PQ186364 GenBank No.). It differed from that of *Paraharmotrema karinganiense* Dutton and Bullard, 2022 (OL413003 GenBank No.; 1,623 bp) by 100 bp (8%), from *Liolope copulans* (Cohn,1902) Baba, Hosoi, Urabe, Shimazu, Tochimoto, and Hasegawa, 2011 (AB551568 GenBank No.; 1,303 bp) by 46 bp (4%), and from *Harmotrema laticaudae* Yamaguti, 1933 (OL413009 GenBank No.; 1,303 bp) by 44 bp (4%). The phylogenetic analysis recovered Liolopidae as monophyletic, with *P. karinganiense* sister to the remaining liolopids. *Dracovermis* (represented only by our sequence of *D. occidentalis*) was recovered sister to the clade comprising *H. laticaudae* and *L. copulans* (Fig. 3).

## Discussion

We accept 3 nominal species of *Dracovermis*: *D. occidentalis, D. brayi* Brooks and Overstreet 1978*,* and *D. rudolphii* (Tubangui and Masilungan, 1936) Brooks and Overstreet 1978. We regard *D. nicolli* (Mehra, 1936) Brooks and Overstreet 1978 as *incertae sedis* because it has a pyriform, relatively sharply-tapering anterior body end (vs. the accepted species of *Dracovermis* all having body ends that are equally rounded) and a vitellarium that extends from the cecal bifurcation to the posterior body end (vs. distributing from the level of the acetabulum or from slightly anterior to the acetabulum to the posterior body end in the accepted species of *Dracovermis*). It also has a diminutive oral sucker rather than the well-developed, bowl-shaped oral sucker of accepted *Dracovermis* spp. Based on the combination of these features, *D. nicolli* could represent a new genus of Liolopidae.

Dutton et al. (2022) stated that previous studies lacked adequate taxon sampling to assess liolopid phylogenetic relationships and liolopid-vertebrate cophyly. That situation has not changed because currently only 4 species (1 from each genus) have been sequenced (Fig. 3). The tree recovered herein again is equivocal regarding vertebrate-liolopid cophyly because *Dracovermis* is sister to the liolopids infecting snakes and amphibians.

We examined adult alligators, 20 from eastern South Carolina (Crawl Creek, Santee River), 8 from southern Alabama (Mobile Bay), 5 from Pascagoula River (Mississippi) and Mississippi Sound (northern Gulf of Mexico) (SAB, unpublished data) as well as 15 juvenile alligators from the type locality for *D. occidentalis* (Rockefeller Wildlife Refuge, Cameron Parish, Grand Chenier, Louisiana). Of those, only one adult alligator from Mobile Bay was infected. Scott et al. (1997), the only other report of *D. occidentalis* since 1978, documented infections (prevalence = 1 of 25; intensity = 2) of *D. occidentalis* in adult alligators from the Trinity River, Texas. Their analysis showed that infection by *D. occidentalis* was significantly higher among adult alligators (no juvenile alligator was infected). The present publication is the first to examine a large number of juvenile alligators for a liolopid infection. Our data and that of Scott et al. (1997) indicate that juvenile alligators lack infections of *D. occidentalis*. We suspect that this could be related to a dietary shift of adult alligators eating large aquatic vertebrates (e.g., fishes, reptiles, mammals) (Delany 1990; Delany et al. 1999; Platt et al. 1990; Scott et al. 1997; Taylor 1986; Wolfe et al. 1987) that could be the second intermediate host for *D. occidentalis*. Regardless of the mechanism(s), our results clearly show that alligators from different areas have different parasites and that certain parasite species can be rare across the known geographic range of the alligator.

## Data Availability

No datasets were generated or analysed during the current study.
